# A Light-Controlled Allosteric Modulator Unveils a Role for mGlu_4_ Receptors During Early Stages of Ischemia in the Rodent Cerebellar Cortex

**DOI:** 10.3389/fncel.2018.00449

**Published:** 2018-11-27

**Authors:** Simon Bossi, Romain Helleringer, Micaela Galante, Ester Monlleó, Ana Trapero, Xavier Rovira, Hervé Daniel, Amadeu Llebaria, Heather McLean

**Affiliations:** ^1^Pharmacologie et Biochimie de la Synapse, Institut des Neurosciences Paris-Saclay, Université Paris-Saclay, Université Paris-Sud - CNRS, UMR 9197, Orsay, France; ^2^MCS, Laboratory of Medicinal Chemistry, Institute for Advanced Chemistry of Catalonia (IQAC-CSIC), Barcelona, Spain; ^3^Molecular Photopharmacology Research Group, The Tissue Repair and Regeneration Laboratory, University of Vic – Central University of Catalonia, Vic, Spain

**Keywords:** presynaptic metabotropic glutamate receptor 4, allosteric modulation, photo-pharmacology, cerebellar cortex, oxygen glucose deprivation

## Abstract

Metabotropic glutamate receptors (mGlus) are G Protein coupled-receptors that modulate synaptic transmission and plasticity in the central nervous system. Some act as autoreceptors to control neurotransmitter release at excitatory synapses and have become attractive targets for drug therapy to treat certain neurological disorders. However, the high degree of sequence conservation around the glutamate binding site makes the development of subtype-specific orthosteric ligands difficult to achieve. This problem can be circumvented by designing molecules that target specific less well conserved allosteric sites. One such allosteric drug, the photo-switchable compound OptoGluNAM4.1, has been recently employed to reversibly inhibit the activity of metabotropic glutamate 4 (mGlu_4_) receptors in cell cultures and *in vivo*. We studied OptoGluNAM4.1 as a negative modulator of neurotransmission in rodent cerebellar slices at the parallel fiber – Purkinje cell synapse. Our data show that OptoGluNAM4.1 antagonizes pharmacological activation of mGlu_4_ receptors in a fully reversible and photo-controllable manner. In addition, for the first time, this new allosteric modulator allowed us to demonstrate that, in brain slices from the rodent cerebellar cortex, mGlu_4_ receptors are endogenously activated in excitotoxic conditions, such as the early phases of simulated cerebellar ischemia, which is associated with elevated levels of extracellular glutamate. These findings support OptoGluNAM4.1 as a promising new tool for unraveling the role of mGlu_4_ receptors in the central nervous system in physio-pathological conditions.

**HIGHLIGHTS**

- The photo-switchable NAM, OptoGluNAM4.1 is functional on native mGlu_4_ receptors in cerebellar cortical slices.
- mGlu_4_ receptors are activated by endogenous glutamate released during simulated ischemia.

## Introduction

Glutamate is the main excitatory transmitter in the central nervous system and exerts its fast actions through ionotropic receptors. This neurotransmitter can also modulate synaptic activity by way of metabotropic G protein-coupled receptors that are expressed on both pre- and postsynaptic membranes. Metabotropic glutamate receptors (mGlus), are involved in several physiological and pathological processes, and are a particularly interesting target for the development of novel therapeutic drugs (Conn and Pin, 1997; Nicoletti et al., 2011).

mGlu receptors assemble as homo- or heterodimers (Doumazane et al., 2011; Kammermeier, 2012) in the plasma membrane. Excepting mGlu_6_, the other group III receptor subtypes (mGlu_4_,_7_, and _8_) are predominantly located on presynaptic terminals, where they inhibit glutamate (autoreceptors) and/or GABA (heteroreceptors) synaptic transmission (Ferraguti and Shigemoto, 2006; Mercier and Lodge, 2014). Most group III sub-types are thought to be coupled to a G protein complex that negatively regulates adenylate cyclase. However, we have recently shown that at least in the cerebellar cortex, presynaptic mGlu_4_ receptors located on Parallel Fibers (PFs) reduce glutamatergic transmission through a mechanism that involves the protein Gα_q_ – PLC pathway (Abitbol et al., 2012; Chardonnet et al., 2017). Similar results have also been obtained for mGlu_7_ in cultured cerebellar granular neurons (Perroy et al., 2000) supporting the idea that the signaling pathways of group III mGlu receptors are not yet fully understood.

Group III mGlu receptors clearly play a role in central nervous system functions as attested by the fact that murine models lacking mGlu_4_, _7_ or _8_ have identifiable phenotypes (Pekhletski et al., 1996; Hölscher et al., 2004, 2005; Goddyn et al., 2015). In *ex vivo* slice preparations obtained from various brain regions, pharmacological activation of presynaptic Group III mGlu receptors by large-spectrum orthosteric agonists such as L-(+)-2-amino-4-phosphonobutyric acid (L-AP4) leads to a reduction in glutamate release (Mercier and Lodge, 2014). However, apart from a few studies showing that these presynaptic receptors can be activated by trains of high frequency afferent stimulation (Chen et al., 2002; Lorez et al., 2003; Cosgrove et al., 2011; Klar et al., 2015), it has been exceedingly difficult to clearly demonstrate a role for these receptors in regulating endogenously glutamate release. It is now commonly accepted that these receptors might in fact be activated in response to excess glutamate accumulation in the extracellular space and as such function as auto-receptors to negatively regulate synaptic glutamate levels (Billups et al., 2005). Presynaptic group III mGlu receptors could come into play in physio-pathological conditions known to induce massive glutamate release, such as epilepsy (Meldrum, 1994; Löscher, 1998) or ischemia (Kalda et al., 2000; Rossi et al., 2000; Panatier et al., 2004; Domin et al., 2016). Activation of these receptors would limit glutamate release and the ensuing excitotoxicity resulting from excessive activation of postsynaptic ionotropic glutamate receptors.

Among the group III receptors, mGlu_4_ has received recent attention owing to its proposed neuroprotective action and its potential involvement in various neurological disorders such as Parkinson’s disease (Beurrier et al., 2009), autism (Becker et al., 2014), and cerebellar ataxia (Power and Empson, 2014). The role of mGlu_4_ in maintaining the homeostasis of extracellular glutamate levels during glutamate excitotoxicity or focal ischemia has been documented (Bruno et al., 2000; Moyanova et al., 2011), but never in the cerebellum.

The cerebellum is involved in fine motor control and excitotoxicity resulting from an ischemic insult can have important consequences on cerebellar function. Purkinje cells (PCs) are GABAergic output neurons located in the cerebellar cortex and are highly involved in motor coordination. These cells are extremely vulnerable to ischemia (Brasko et al., 1995; Welsh et al., 2002; Ardeshiri et al., 2006) and respond to excess extracellular glutamate associated with this condition by a strong anoxic depolarizing current (Hamann et al., 2005; Mohr et al., 2009). Given that mGlu_4_ receptors are present on PFs that innervate both PCs (Daniel and Crepel, 2001; Abitbol et al., 2008) and molecular layer interneurons (Zhang and Linden, 2009; Chardonnet et al., 2017), it seems important to investigate a potential neuroprotective role of these receptors during physio-pathological conditions associated with elevated levels of extracellular glutamate.

Better understanding of the physiological role of mGlu_4_ receptors and the assessment of their therapeutic potential relies on the ability to modulate mGlu_4_ activity in a selective and temporally controlled manner. Since orthosteric ligands that target the highly conserved Venus Fly Trap ligand binding domain of the receptor do not readily discriminate among mGlu subtypes, efforts have been made to develop allosteric ligands that selectively interact with each receptor sub-type. Recently, a photo-pharmacology based approach (Lerch et al., 2016) to mGlu receptor allosteric ligands has produced new molecular tools bringing together both selectivity and real-time control by using light (Pittolo et al., 2014; Zussy et al., 2016). One of these molecules, OptoGluNAM4.1, a group III negative allosteric modulator (NAM), that targets both mGlu_4_ and mGlu_7_ receptors has been recently synthesized and tested (Rovira et al., 2016). OptoGluNAM4.1, an azobenzene-containing compound that isomerizes under blue-light/dark cycles, has proven to be effective in the dynamic photo-control of mGlu_4_ receptor activity, first in antagonizing mGlu_4_ receptor activity in transfected cultured HEK293 cells, second in inducing behavioral changes in free-swimming zebrafish, and finally in a murine model of inflammatory chronic pain (Rovira et al., 2016). In the present study, we have analyzed the effects of OptoGluNAM4.1 on the regulation of excitatory glutamatergic neurotransmission at PF-PC synapses in the rodent cerebellum by mGlu_4_ receptors, which are the only group III mGlu receptors functional at the PF-PC synapse (Abitbol et al., 2012). Our results show that OptoGluNAM4.1 can be temporally controlled in cerebellar slices where it antagonizes mGlu_4_ receptor activation in the dark, an effect that can be reversed in the presence of blue light. Furthermore, this new and selective pharmacological tool has allowed us, for the first time in the cerebellum, to show that mGlu_4_ receptors are activated during the early stages of cerebellar ischemia simulated using a protocol of Oxygen and Glucose Deprivation (OGD) (Rossi et al., 2000; Helleringer et al., 2017).

## Materials and Methods

### Animals

Animals (rats and mice) were housed with *ad libitum* access to food and water at 22–23°C in a standard 12 h light–dark cycle. Animal care and euthanasia procedures are in accordance with European legislation 2010/63EU Council Directive Decree) and following Annex IV of the French Decree (February 1st 2013) establishing the guidelines for euthanasia. Experimental protocols were approved by the Animal Welfare body of our Institution (Institut des Neurosciences, NeuroPSI). All efforts were made to minimize animal suffering and to reduce the number of animals used in this study. Sprague Dawley rats and C57BL/6 wild-type (WT) mice came from Janvier Laboratories (Le Genest-St-Isle, France). To generate mutant mice lacking the mGlu_4_ receptor, Grm4^+/-^ (B6.129-Grm4tm1Hpn/J) animals, produced on a C57BL/6 background, were purchased from Jackson Laboratories (Bar Harbor, United States), with Charles River Laboratories (Saint Germain sur l’Arbresle, France) as the international import and distribution agent. The Grm4-/- offspring of heterozygotes were used to establish colonies of Grm4-/- mice, referred to as mGlu_4_ knock out (mGlu_4_^-/-^) mice. Only male animals (rats and mice) between 3 and 4 weeks of age were used in experiments.

### Preparation of Cerebellar Slices

Male Sprague-Dawley rats (21–26 days) and C57BL/6 WT and knockout mGlu_4_ receptor mice (21–27 days) were anesthetized with 2-Bromo-2-Chloro-1,1,1-Trifluoroethane and then decapitated. Sagittal slices (250 μm thick) were cut from the cerebellar vermis in ice-cold oxygenated saline solution (<1°C) with a vibratome Microm HM 650V (Microm Microtech, France). The saline solution contained (in mM): NaCl, 138.6; KCl, 3; NaHCO_3_, 24; KH_2_PO_4_, 1.15; MgSO_4_, 1.15; CaCl_2_, 2; glucose, 10 and was gassed with 95% O_2_ and 5% CO_2_ (osmolarity, 330 mosmol/L; pH, 7.35). For electrophysiological recording, slices were transferred to a chamber on an upright microscope (Zeiss, Le Peck, France) and perfused at a rate of 2 mL per minute with this same oxygenated saline solution, supplemented with the GABA_A_ receptor antagonist, GABAzine (5 μM). Pharmacological agents were applied to cerebellar slices by direct addition to the saline solution at the desired concentration just before use.

We performed a series of experiments using an Oxygen Glucose Deprivation (OGD) protocol to mimic certain brain conditions associated with an ischemic insult. In this case, we used the same saline solution described above, except that glucose was replaced by the same amount of sucrose and the solution was gassed with 95% N_2_ and 5% CO_2_.

### Electrophysiology

All recordings were performed at 30–32°C. Purkinje cell (PC) somas were visualized using Nomarski optics and a 63X water-immersion objective. Whole-cell patch-clamp recordings of PCs were performed with an Axopatch-1D amplifier (Axon Instruments, Foster City, United States). Patch pipettes (5–6 MΩ, borosilicate glass) were filled with an intracellular solution of the following composition (in mM): K-gluconate, 140; KCl 6; HEPES, 10; EGTA, 0.75; MgCl_2_, 1; Na-GTP, 0.4; Na_2_-ATP, 4; pH adjusted to 7.3 with KOH; 300 mosmol/L. PCs were voltage-clamped at -60 mV and junction potentials were corrected. Parallel fibers (PFs) were stimulated every 6 s (0.17 Hz) with a glass saline-filled monopolar electrode placed at the surface of the slice, in the lower half of the molecular layer, to evoke excitatory postsynaptic currents in PCs (PF-EPSCs). PF-EPSCs were filtered at 5 kHz, digitized on line at 20 kHz, and analyzed on and off-line with Elphy (G. Sadoc, Gif-sur-Yvette, France), Igor (Wavemetrics, United States), and Clampfit (Axon Instruments, United States) software. Series resistance was partially compensated (60–75%) as previously described by Llano et al. (1991). Recordings were terminated if this resistance increased by more than 20% of the initial value.

Throughout the experiment, PCs were clamped at -60 mV but PF-EPSCs were elicited on a 10 mV hyperpolarizing voltage step, which allowed monitoring of passive membrane properties (cell capacitance and membrane resistance). Only cells with stable values of these parameters (less than 20% variation) were retained for analysis. PF-EPSCs were evoked with pairs of stimuli of the same intensity, with an inter-stimulus interval of 40 ms. Paired-pulse ratio (PPR) values (Atluri and Regehr, 1996, 1998) were calculated on-line as the ratio of the amplitude of the second evoked EPSC over the first, and plotted against time. Corresponding PPR values in individual plots were then averaged for all cells recorded, to obtain the plot of mean PPF values over time in control and test conditions.

### Pharmacology

mGlu_4_ receptors were pharmacologically activated by bath application of the group III mGlu receptor orthosteric agonist, L-AP4 (10 μM). We chose to employ non-saturating concentrations of this agonist (Abitbol et al., 2008) to evaluate the putative effects of OptoGluNAM4.1 (50 μM) on mGlu_4_ receptor activity, since changes in ligand affinity induced by allosteric modulation of mGlu_4_ receptors should be readily discernable at non-saturating ligand concentrations (Conn et al., 2009).

OptoGluNAM4.1 is a photo-isomerizable azobenzene molecule that can be switched from its active (*trans*) to its inactive (*cis*) configuration by blue light. Care was taken to limit the amount of ambient light that reached the recording chamber. Experiments were performed in a dark room and the Faraday cage was reinforced with black panels positioned on all sides of the cage to limit light infiltration. OptoGluNAM4.1 is maintained in its active *trans* configuration in the dark. When the molecule is illuminated with blue light (420–460 nm), it photo-isomerizes to its inactive *cis* configuration. To inactivate this compound, we used an LED light source (Collimated LED Light Sources, M455L3-C4, Royal Blue 455 ± 18 nm; 430 mW total beam power; Thorlabs, United States), that directly illuminated the recording chamber. In all experiments, slices were exposed to both blue light and dark conditions, whether we applied L-AP4 or OGD alone or in the presence of OptoGluNAM4.1. However, each slice was exposed to only one L-AP4 or OGD application.

**Figure:**
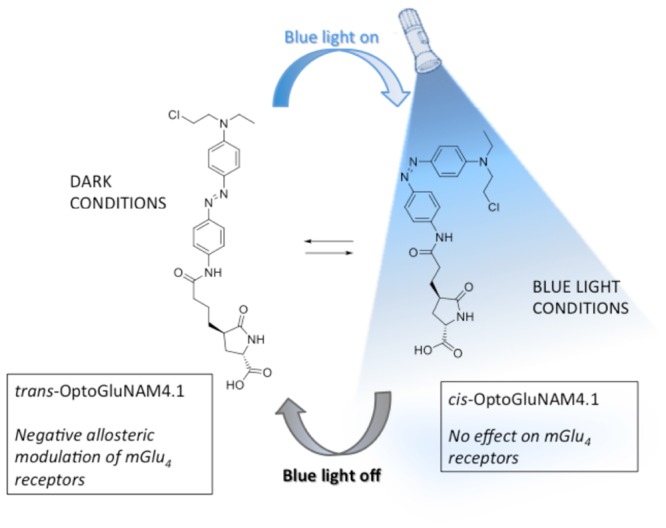


L-AP4 (L-(+)-2-amino-4-phosphonobutyric acid) and MSOP [(RS)-α-methylserine-O-phosphate] were purchased from Tocris (Illkirch, France). OptoGluNAM4.1 was synthesized in the MCS laboratory of the Institute for Advanced Chemistry of Catalonia (for details see Rovira et al., 2016). GABAzine (SR95531) was purchased from Abcam. All drug stocks were prepared in distilled water except for OptoGluNAM4.1 that was prepared in DMSO (dimethylsulfoxide). The final concentration of DMSO was 0.1%. Drug stocks were kept at -20°C until use.

### Data Analysis and Statistics

To analyze the effects of L-AP4, active and inactive OptoGluNAM4.1 and MSOP on PF–EPSCs, the amplitude of evoked currents was calculated on line and normalized to control amplitudes. Unless otherwise stated, control amplitude was that measured before any drugs were applied. The average percent depression associated with the L-AP4 effect was calculated over a 3 min period at the peak of the L-AP4 effect. The OGD protocol lasted for 20 min. We analyzed the effect of our compounds on the amplitude of evoked synaptic currents between 5 and 10 min into the protocol, just before PCs developed the large inward depolarizing anoxic current (Hamann et al., 2005).

All data are presented as mean ± S.E.M. Statistical significance was assessed for normally distributed data by an unpaired or paired (as indicated) Student’s *t*-test, with *p* < 0.05 (two-tailed) considered as significant. The similarity of variances between each group of results was tested using an *F*-test. In cases of variance differences, a Welch *T*-test was used to evaluate statistical significance. « n » indicates the number of cells included in the statistics. When the sample number was less than 6, a Mann and Whitney test was used to assess statistical significance.

Analysis of drug effects over time was effected by a one-way analysis of variance (ANOVA). A repeated measures ANOVA was used to establish significance for an overall drug effect. This was followed by *post hoc* analysis, Student’s *t*-tests, for individual comparisons where appropriate. A *P*-value <0.05 was considered statistically significant.

## Results

In the cerebellar cortex, mGlu_4_ receptors are expressed in the presynaptic active zone of synapses between glutamatergic PF and their targets (Mateos et al., 1998, 1999; Corti et al., 2002). mGlu_4_ receptors are the only functional group III subtype found on PF terminals (Abitbol et al., 2008) making the cerebellar cortex an ideal model for functional studies on these receptors. In this study, we tested the ability of OptoGluNAM4.1, a photo-isomerizable NAM of mGlu_4_, to modulate the activity of these native receptors at PF–PC synapses. We tested the active and inactive form of this molecule to investigate the mGlu_4_ – mediated inhibition of PC excitatory postsynaptic currents (EPSCs) evoked by electrical stimulation of PFs (PF–EPSCs) during concomitant activation of these receptors by the group III mGlu receptor orthosteric agonist, L-AP4 (10 μM) or during the early phase of simulated ischemia.

### Blue Light Has No Effect on Evoked Synaptic Responses or Pharmacological Activation of mGlu_4_ Receptors at PF-PC Synapses

**FIGURE 1:**
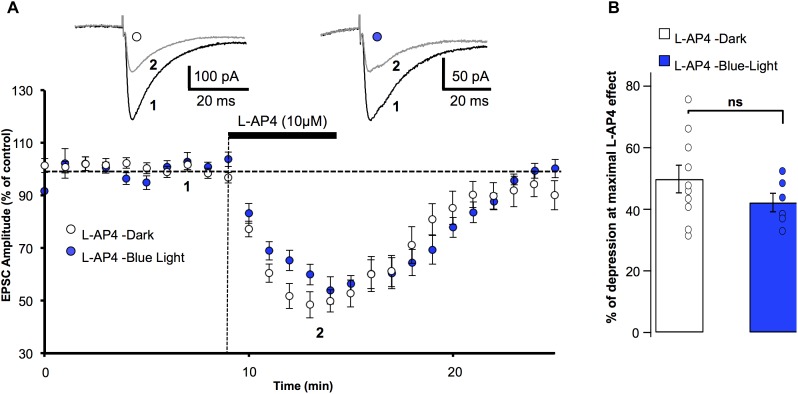
Blue light has no effect on mGlu_4_ receptor modulation of evoked EPSC amplitude in rats. **(A)** Plot of normalized amplitudes of PF-evoked excitatory postsynaptic currents (PF-EPSCs) as a function of time before, during and after bath application of 10 μM L-AP4 (horizontal black bar) under 2 experimental conditions: in the dark (white circles, *n* = 10), and under blue (455 nm) light (blue circles, *n* = 6). For this and the following figures, each point on the graph is the average PF-EPSC amplitude for all cells in the group. Upper traces are averaged (5 consecutive trials) recording traces from one experiment taken before (1) and during (2) L-AP4 application, under the two experimental conditions. **(B)** The bar graph shows the inhibitory effect of L-AP4 as percent depression in PF-EPSC amplitude calculated over a 3 min period at the peak of the L-AP4 effect in the dark (white) and under blue light (blue). Data are means ± SEM.

Given that OptoGluNAM4.1 was switched from its active to its inactive form by blue light (Rovira et al., 2016), it was important to know whether persistent illumination of the slice with this light influenced synaptic transmission or modified the L-AP4 effect on PF-EPSCs. Figure 1A shows the effect of bath application of L-AP4 on PF–EPSC amplitude in the dark and in the presence of blue light. L-AP4 effects were reversible under both conditions with PF-EPSC amplitude attaining control (pre-L-AP4) levels within 10 min following the end of the L-AP4 application. Moreover, blue light illumination had no major effect on the time course of this recovery. We quantified the effect of L-AP4 on PF-EPSC amplitude over 3 min at the peak of the L-AP4-induced depression (Figure 1B). In the dark, L-AP4 reduced PF-EPSCs by 49.8 ± 4.5% compared to control (pre-L-AP4) amplitude (*n* = 10) while with the blue light on, PF-EPSCs were depressed by 42.2 ± 3% (*n* = 6). These values, that are summarized in Figure 1B, are not significantly different (*p* > 0.2).

Keeping in mind that mGlu_4_ is the only group III receptor subtype functional at the PF-PC synapse (Abitbol et al., 2012), our results confirm that the L-AP4 effect on PF-EPSCs depends on mGlu_4_ receptor activation, and show that exposing the cerebellar slices to blue light does not significantly alter the depressive effect of L-AP4 on PF-EPSCs.

### Active OptoGluNAM4.1 Antagonizes L-AP4 Actions at the PF-PC Synapse: Effect on PF-EPSC Amplitude

**FIGURE 2:**
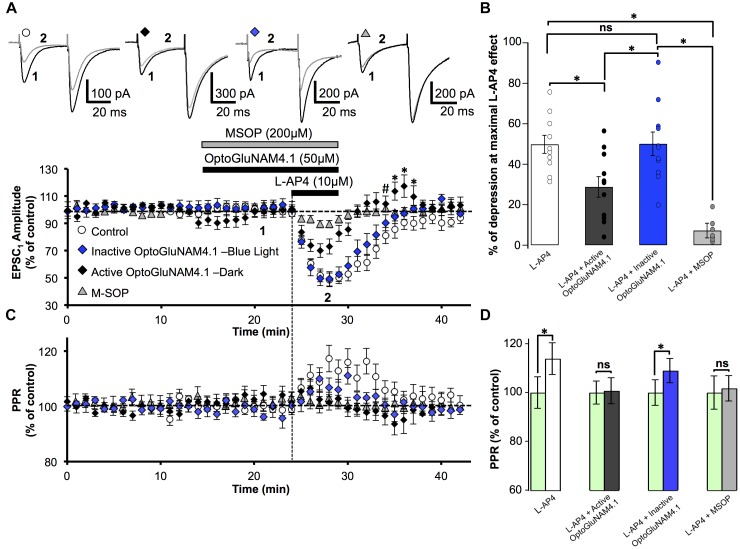
Active OptoGluNAM4.1 reduces mGlu_4_ receptor-induced depression of evoked EPSC amplitude in rats. **(A)** Plot of normalized amplitude of the first PF-EPSC recorded as a function of time before, during, and after 10 μM L-AP4 application (horizontal black bar) alone (white circles, *n* = 10), in the presence of inactive *cis*-OptoGluNAM4.1 (blue diamonds, *n* = 11), in the presence of active *trans*-OptoGluNAM4.1 (black diamonds, *n* = 11), and in the presence of MSOP (gray triangles, *n* = 7). After washout of L-AP4 and active trans-OptoGluNAM4.1, PF-EPSP amplitude was transiently increased compared to control values (# no significant difference from control, ^∗^ significant difference from control). Upper traces are averaged (5 consecutive trials) recording traces from one experiment taken before (1) and during (2) L-AP4 application, under the four experimental conditions. **(B)** The bar graph shows the inhibitory effect of L-AP4 as percent depression in PF-EPSC amplitude calculated over a 3 min period at the peak of the L-AP4 effect under the four experimental conditions mentioned above. **(C)** Plot of normalized values of the paired-pulse ratio (PPR) shows transient increases in PPR during L-AP4-mediated depression of PF-EPSC amplitude. **(D)** The bar graph represents the normalized PPR measured during a 5 min period at the peak of the L-AP4 effect as a percentage of control (pre-L-AP4 application, green bar) with: L-AP4 alone (white), L-AP4 in the presence of inactive *cis*-OptoGluNAM4.1 (blue), L-AP4 in the presence of active *trans*-OptoGluNAM4.1 (black) and L-AP4 in the presence of MSOP (gray). Means ± SEM. (^∗^*p* < 0.05).

We then studied the capacity of the photo-switchable NAM, OptoGluNAM4.1, to modulate the activity of pharmacologically activated mGlu_4_ receptors at PF–PC synapses. OptoGluNAM4.1 is photo-isomerized to its inactive *cis* state in the presence of blue light, while in the dark, this molecule undergoes spontaneous thermal relaxation to regain and maintain its active *trans* configuration. Bath application of either the active (*trans*) or inactive (*cis*) isomer of OptoGluNAM4.1 (50 μM) alone had no significant effect on the amplitude of PF–EPSCs (Figure 2A). With co-application of active *trans*-OptoGluNAM4.1 and L-AP4, the PF–EPSC amplitude was reduced by 28.8 ± 5.1% (*n* = 11), a value significantly lower than that observed with L-AP4 alone (49.8 ± 4.5%, *n* = 10, *p* < 0.01) (Figure 2B). In contrast, in the presence of the inactive *cis* isomer of OptoGluNAM4.1, L-AP4 reduced the average PF-EPSC amplitude by 50.1 ± 5.8% (*n* = 11), a value comparable to that obtained with L-AP4 alone (*p* > 0.9). These data show that the active *trans* isomer of OptoGluNAM4.1 attenuates the L-AP4 induced depression of PF–EPSCs. As a control, we performed these same experiments in the presence of a broad-spectrum group III receptor antagonist, MSOP (200 μM). Bath application of MSOP alone had no effect on the amplitude of PF-EPSCs (Figure 2A). In the presence of MSOP, L-AP4 reduced the PF–EPSC amplitude by only 7.2 ± 3.6% of pre-L-AP4 values (*n* = 7), a value significantly lower than that obtained in the presence of L-AP4 alone (*p* < 0.001, Figure 2B). It is notable that the effect of *trans*-OptoGluNAM4.1 on PF mGlu_4_ receptors is weaker than that of MSOP. This is not entirely surprising since Rovira et al. (2016) also found that in cell assays, OptoGluNAM4.1 only partially blocked L-AP4 induced mGlu_4_ receptor activity.

### OptoGluNAM4.1 Acts on Presynaptic mGlu_4_ Receptors Located on PF Terminals: Effect on PPR

PC EPSCs evoked by paired electrical stimulation of PFs (40 ms interval, described in Materials and Methods) showed a high degree of paired-pulse facilitation (PPF, traces Figure 2A). This form of short-term plasticity is characteristic of synapses with low transmitter release probability (Atluri and Regehr, 1996, 1998) and depends, at least in part, on incomplete calcium clearance in presynaptic terminals after the first stimulus, leading to increased transmitter release with the second stimulus (Zucker and Regehr, 2002). Moreover, experimental paradigms that result in changes in the paired pulse ratio (PPR) are indicative of events that occur at the pre-synapse.

Bath application of L-AP4 alone significantly increased normalized PPR values by 13.9%, from 100 ± 6.4% (pre-L-AP4) to 113.9 ± 6.5% (*p* < 0.01) (Figures 2C,D). When L-AP4 was applied in the presence of the group III antagonist, MSOP, there was no significant change in PPR values compared to those in the presence MSOP alone (Figures 2C,D). Curiously, when L-AP4 was applied in the presence of active *trans*-OptoGluNAM4.1, we observed no significant change in PPR values compared to those in the presence of *trans*-OptoGluNAM4.1 alone (Figures 2C,D). Given that *trans*-OptoGluNAM4.1 only partially blocked the L-AP4 induced decrease in PF-EPSC amplitude (Figures 2A,B), one might expect to see a slight, albeit not necessarily significant increase in PPR under these experimental conditions.

In the presence of inactive *cis*-OptoGluNAM4.1, L-AP4 significantly increased the PPR by 9.0% (100 ± 5% in inactive *cis*-OptoGluNAM4.1 alone and 109 ± 5% in inactive *cis*-OptoGluNAM4.1 + L-AP4, *p* < 0.05) (Figures 2C,D). This increase was comparable (*p* > 0.05) to that observed with L-AP4 alone. These results confirm that OptoGluNAM4.1 is a photo-switchable NAM of native mGlu_4_ receptors and that this molecule acts on mGlu_4_ receptors located at the pre-synapse.

### Active Trans-OptoGluNAM4.1 Alters the Recovery Profile of the L-AP4 Depressant Effect

Close inspection of Figure 2A reveals that following co-application of L-AP4 and active *trans*-OptoGluNAM4.1 the amplitude of PF-EPSCs not only recovered more quickly than with L-AP4 alone, but PF-EPSCs were transiently potentiated before returning to baseline values. This finding was surprising, particularly since active *trans*-OptoGluNAM4.1 alone had no effect on PF-EPSC amplitude. We thus performed a one-way ANOVA analysis on the three experimental groups (L-AP4, L-AP4 + *trans*-OptoGluNAM4.1 and L-AP4 + *cis*- OptoGluNAM4.1) during this period of transient potentiation (Figure 2A). Five minutes after the end of L-AP4 + OptoGluNAM4.1 application, PF-EPSC amplitude was not significantly different between the three experimental groups [*F*(2, 26) = 3.201, *p* > 0.05, Figure 2A,^#^]. However, between 6 and 8 min after the end of the L-AP4 + *trans*-OptoGluNAM4.1 application (Figure 2A,^∗^), a repeated measures ANOVA analysis showed significant differences between these three groups [6 min after *F*(2, 25) = 4.482, *p* < 0.05; 7 min after *F*(2, 24) = 3.876, *p* < 0.05; 8 min after *F*(2, 24) = 3.968, *p* < 0.05].

Thus, it appears that active *trans*-OptoGlu4.1 modulates mGlu_4_ actions on PF-EPSC amplitude not only during the L-AP4 effect, but also during early recovery. This potentiation of PF-EPSCs post L-AP4 application was not observed following co-application of L-AP4 and MSOP (Figure 2A).

### Active OptoGluNAM4.1 Antagonizes L-AP4 Pharmacological Activation of mGlu_4_ Receptors in WT Mice, but Has No Effect in mGlu_4_^-/-^ Mutant Mice

**FIGURE 3:**
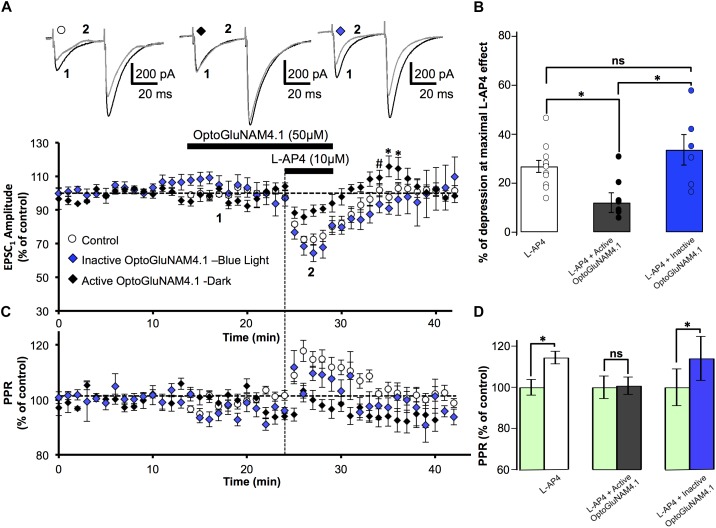
Active OptoGluNAM4.1 reduces mGlu_4_ receptor-induced depression of evoked EPSC amplitude in wild-type mice. **(A)** Plot of normalized amplitude of the first PF-EPSC recorded as a function of time before, during, and after 10 μM L-AP4 application (horizontal black bar) alone (white circles, *n* = 12), in the presence of inactive *cis*-OptoGluNAM4.1 (blue diamonds, *n* = 6), and in the presence of active *trans*-OptoGluNAM4.1 (black diamonds, *n* = 9). After washout of L-AP4 and active trans-OptoGluNAM4.1, PF-EPSP amplitude was transiently increased compared to control values (# no significant difference from control, ^∗^ significant difference from control). Upper traces are averaged (5 consecutive trials) recording traces from one experiment taken before (1) and during (2) L-AP4 application, under the three experimental conditions. **(B)** The bar graph shows the inhibitory effect of L-AP4 as percent depression in PF-EPSC amplitude calculated over a 3 min period at the peak of the L-AP4 effect under the three experimental conditions mentioned above. **(C)** Plot of normalized values of the PPR shows transient increases in PPR during L-AP4-mediated depression of PF-EPSC amplitude. **(D)** The bar graph represents the normalized PPR measured during a 5 min period at the height of the L-AP4 effect as a percentage of control (pre-L-AP4 application, green bar) with: L-AP4 alone (white), L-AP4 in the presence of active *trans*-OptoGluNAM4.1 (black) and L-AP4 in the presence of inactive *cis*-OptoGluNAM4.1 (blue). Means ± SEM. (^∗^*p* < 0.05).

To confirm that in our model OptoGluNAM4.1 acts specifically on mGlu_4_ receptors, we employed one of the most common strategies to complement our neuropharmacology, we compared the effects of the NAM on mGlu_4_ receptor activity in wild type (WT) mice and mice in which the mGlu_4_ gene has been invalidated (mGlu_4_^-/-^). In cerebellar slices obtained from WT mice, bath application of L-AP4 alone reversibly reduced the amplitude of PF–EPSCs by 26.9 ± 2.4%, *n* = 12) (Figure 3A). As previously documented (Abitbol et al., 2008), the L-AP4 effect in mice was less pronounced than that observed in rats (cf Figure 2A). Even so, active *trans*-OptoGluNAM4.1, co-applied with L-AP4, significantly attenuated the L-AP4 effect on PF-EPSC amplitude (12.1 ± 4.0% reduction, *n* = 9, *p* < 0.05) (Figures 3A,B). As expected, in the presence of inactive *cis*-OptoGluNAM4.1, the L-AP4 effect was comparable to that observed with L-AP4 alone: reduction in PF–EPSC amplitude of 33.6 ± 6.2% in L-AP4 plus *cis*-OptoGluNAM4.1 (*n* = 6) versus 26.9 ± 2.4% in L-AP4 alone, *p* > 0.2 (Figures 3A,B).

As was seen in rats, the depressant effect of L-AP4 on PF-EPSC amplitude in WT mice was accompanied by an increase in the PPR (Figures 3C,D) that was abolished by co-application of active *trans*-OptoGluNAM4.1 (*n* = 9, *p* < 0.001, Figure 3D). Moreover, when L-AP4 was applied in the presence of inactive *cis*- OptoGluNAM4.1 (*n* = 6), the associated PPR was increased by 14% (100 ± 3.8% in *cis*- OptoGluNAM4.1 alone and 114 ± 10.7% in *cis*-OptoGluNAM4.1 + L-AP4, *p* < 0.05), but was not different to that observed during application of L-AP4 alone (*p* > 0.2, Figure 3D).

As with experiments performed on rats, the L-AP4 induced depression of PF-EPSC amplitude recovered more quickly when L-AP4 was co-applied with *trans*-OptoGluNAM4.1 compared to when L-AP4 was applied alone (Figure 3A). Five minutes after the end of L-AP4 + *trans*-OptoGluNAM4.1 application, PF-EPSC amplitude was not significantly different between the three experimental groups [*F*(2, 24) = 2.712, *p* > 0.05, Figure 3A,#]. In addition, we also observed a transient increase in PF-EPSC amplitude following the end of L-AP4 + *active trans*-OptoGluNAM4.1 application. Significant differences between groups were seen 6 min after [F(2, 24) = 6.986, *p* < 0.05] and 7 min after [*F*(2, 24) = 4.019, *p* < 0.05] the end of application (Figure 3A,^∗^). This transient increase was not associated with a change in PPR (Figure 3C).

**FIGURE 4:**
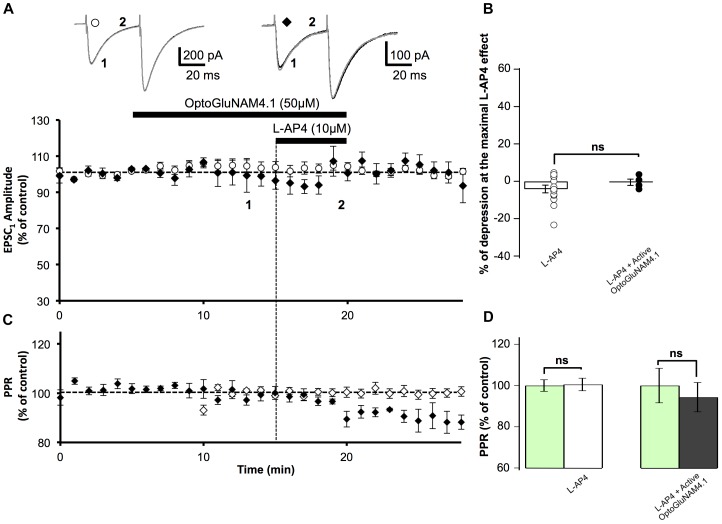
Active OptoGluNAM4.1 has no effect on evoked EPSCs in mGlu_4_^-/-^ mice. **(A)** Plot of normalized amplitude of the first PF-EPSC recorded as a function of time before, during, and after 10 μM L-AP4 application (horizontal black bar) alone (white circles, *n* = 14) and in the presence of active *trans*-OptoGluNAM4.1 (black diamonds, *n* = 4). Upper traces are averaged (5 consecutive trials) recording traces from one experiment taken before (1) and during (2) L-AP4 application, under the two experimental conditions. **(B)** The bar graph shows the inhibitory effect of L-AP4 as percent depression in PF-EPSC amplitude calculated over a 3 min period at the peak of the L-AP4 effect, under the two experimental conditions mentioned above. **(C)** Plot of normalized values of the PPR shows that L-AP4 alone or co-applied with *trans*-OptoGluNAM4.1 has no effect on PPR. **(D)** The bar graph represents the normalized PPR measured during a 5 min period during L-AP4 application as a percentage of control (pre-L-AP4 application, green bar) with: L-AP4 alone (white) and L-AP4 in the presence of active *trans*-OptoGluNAM4.1 (black). Means ± SEM.

In slices obtained from mGlu_4_ KO mice (mGlu_4_^-/-^), bath application of L-AP4 had no effect on either the amplitude (Figures 4A,B) or the PPR (Figures 4C,D) of PF-EPSCs (*n* = 14). These data are consistent with our previously published results (Abitbol et al., 2008). Similarly, active *trans*-OptoGluNAM4.1 affected neither the amplitude nor the PPR of PF–EPSCs when applied alone or with L-AP4 (*n* = 4). Taken together, these data demonstrate that in WT mice, like in rats, OptoGluNAM4.1 in its active *trans* configuration, negatively modulates L-AP4 induced activation of mGlu_4_ receptors, and has no effect in mice lacking these receptors. In addition, in both rats and mice L-AP4 and OptoGluNAM4.1 act on presynaptic mGlu_4_ receptors.

### OptoGluNAM4.1 Reveals a Functional Role for mGlu_4_ Receptors in Rats

Despite their high affinity for glutamate and their strategic location in the pre-synaptic active zone of central nervous system synapses (Mateos et al., 1998, 1999; Corti et al., 2002), it has been exceedingly difficult to demonstrate a role for mGlu_4_ receptors in the regulation of synaptic transmission under conditions of endogenous glutamate release (Lorez et al., 2003). It is likely that very high levels of extracellular glutamate may be required to activate these receptors in acute slice preparations. As such, we employed an experimental protocol of OGD that mimics brain ischemia, and that is associated with a dramatic increase in extracellular glutamate levels in the central nervous system (Rossi et al., 2000; Mohr et al., 2009). We subjected cerebellar slices to 20 min of OGD (see Materials and Methods), followed by a period of recovery in normal, oxygenated bicarbonate buffered saline. In our hands, after 15–20 min of exposure to OGD, PCs developed a large inward current (several nA), that in a few cases returned to control levels after recovery from the ischemic insult. This current, commonly referred to as anoxic depolarization, is typical of PCs that have been exposed to OGD and has been described in detail (Hamann et al., 2005).

**FIGURE 5:**
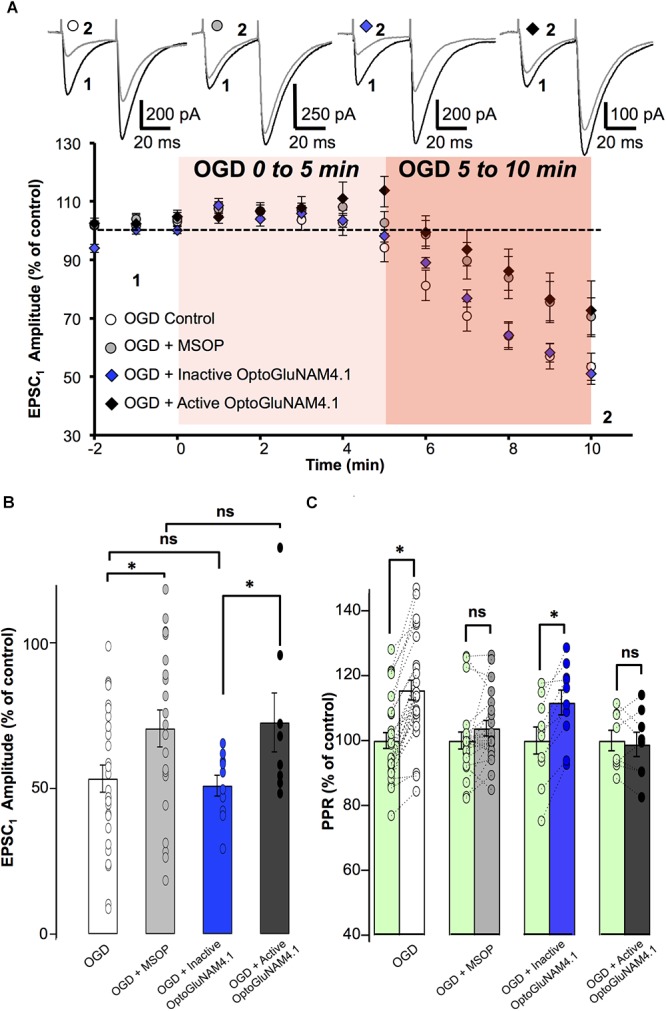
mGlu_4_ activation by endogenous glutamate during Oxygen Glucose Deprivation in rats. **(A)** Plot of normalized amplitudes of the first PF-EPSC recorded as a function of time, before and during the first 10 min of OGD under the following experimental conditions: OGD alone (white circles, *n* = 27), OGD with 200 μM MSOP (gray circles, *n* = 21), OGD with inactive *cis*-OptoGluNAM4.1 (blue diamonds, *n* = 10) and OGD with active *trans*-OptoGluNAM4.1 (black diamonds, *n* = 8). Upper traces are averaged (5 consecutive trials) recording traces from one experiment taken before (1) and 10 min into the OGD protocol (2) for each experimental condition. **(B)** The bar graph shows the percent depression in PF-EPSC amplitude calculated over a 1 min period after 10 min of OGD, under the four experimental conditions mentioned above. **(C)** The bar graph shows the normalized PPR before OGD (green bar) and averaged during the 5–10 min period of OGD for the 4 experimental conditions: OGD alone (white), OGD with MSOP (gray), OGD with inactive *cis*-OptoGluNAM4.1 (blue) and OGD with active *trans*-OptoGluNAM4.1 (black). Means ± SEM. (^∗^*p* < 0.05).

We chose to analyze the effect of OGD on PF–EPSCs during the early phase of the protocol (between 6 and 10 min), before the anoxic depolarizing inward current appeared because once this current developed in PCs, it was difficult to identify and isolate individual PF-EPSPs. Throughout this initial period of OGD, we observed no change in membrane input resistance of the cells retained for analysis. As illustrated in Figure 5A, after 5 min of OGD, PF–EPSC amplitude began to gradually decrease (between 6 and 10 min), reaching 53.4 ± 4.7% of pre-OGD values after 10 min of OGD (*n* = 27, Figure 5B). Moreover, as shown in Figure 5C, in all cells tested, this depression was associated with a significant increase in the PPR (115.0 ± 3.1%; *p* < 0.001), suggesting that during OGD there is a reduction in the probability of glutamate release from PFs. To determine whether group III mGlu receptors are activated during OGD and as such could be at least partly responsible for the OGD-induced depression of PF-EPSC amplitude, we repeated this protocol under three additional experimental conditions, namely, in the presence of MSOP, inactive *cis*-OptoGluNAM4.1, or with active *trans*-OptoGluNAM4.1 (Figure 5).

A one-way ANOVA test revealed no significant difference in PF-EPSC amplitude among the four independent groups 5 min after the onset of simulated ischemia [*F*(3, 62) = 2.12, *p* > 0.05]. In contrast, there were significant differences between the four independent groups 6 min [*F*(3, 62) = 2.923, *p* < 0.05], 7 min [*F*(3, 62) = 3.118, *p* < 0.05], 8 min [*F*(3, 62) = 3.252, *p* < 0.05], 9 min [*F*(3, 62) = 2.870, *p* < 0.05], and 10 min [*F*(3, 62) = 2.905, *p* < 0.05] after OGD onset. The recordings were ceased after 10 min of OGD exposure. In this context, and to evaluate the significant overall effect of the simulated ischemia between the four independent groups, a repeated measure of variance performed between minute 6 and 10 [*F*(3, 286) = 10.97, *p* < 0.05], demonstrated that there is a significant difference between these groups. Considering the similarity of variances between each independent group previously tested using an *F*-test, the ANOVA analysis was followed by Student’s *t*-tests. For each independent group, the OGD-related reduction in PF- EPSC amplitude was measured between 6 and 10 min of OGD exposure.

The orthosteric group III antagonist MSOP significantly attenuated the OGD-related reduction in PF-EPSC amplitude by 18.4% (16.3 ± 6.2% in MSOP, *n* = 21, versus 34.7 ± 4.5% in OGD alone, *n* = 27, *p* < 0.05) (Figure 5A). Furthermore, in the presence of MSOP, OGD had no effect on the PPR (Figure 5C). These results show that presynaptic group III mGlu receptors can be activated during the early phase of OGD.

To confirm that mGlu_4_ receptors are involved in the OGD response, we tested the effect of OptoGluNAM4.1 on PF-EPSC amplitude during the early phase of OGD. As shown in Figure 5A, in the presence of active *trans*-OptoGluNAM4.1, PF-EPSC amplitude was reduced by only 14.3 ± 7.1% (*n* = 8), a value significantly smaller than that observed in OGD alone (*p* < 0.05). Moreover, no changes in PPR were observed during OGD under these experimental conditions (98.8 ± 3.7% of control, Figure 5C). These data show for the first time that PF mGlu_4_ receptors are activated during the first phase of OGD whereby limiting glutamate release in this pathophysiological condition.

If mGlu_4_ receptors do in fact have a neuroprotective role, one might expect that their activation might influence not only the amount of glutamate released during OGD, but also the time to onset of the OGD effect on synaptic transmission. OGD-dependent decreases in PF–EPSC amplitude become apparent after 6 min of exposure under all our experimental conditions. However, the precise time to onset of this response depended on the level of activation of mGlu_4_ receptors. Reducing the activity of these receptors, either with the orthosteric group III antagonist MSOP or active *trans*-OptoGluNAM4.1, increased the time to onset of the OGD–induced depression of PF–EPSC amplitude compared to control conditions. When OGD was applied alone (*n* = 27), or in the presence of inactive *cis*-OptoGluNAM4.1 (*n* = 10), PF–EPSCs showed decreases in amplitude 5.9 ± 0.48 and 5.9 ± 0.46 min, respectively, after the beginning of the episode (Supplementary Figure S1). When mGlu_4_ receptor activity was inhibited, either by active *trans*-OptoGluNAM4.1 (*n* = 8) or by MSOP (*n* = 21), the onset-latency of the OGD response was prolonged to 8 ± 0.6 min in *trans*-OptoGluNAM4.1 and to 8 ± 0.47 min in MSOP. These values are significantly different to those measured in OGD alone (*p* < 0.05). As such, not only does active *trans* OptoGluNAM4.1 reduce the OGD effect on PF–EPSC amplitude and PPR, but it also delays the time to onset of these responses. Taken together, these results demonstrate that mGlu_4_ receptors are activated by endogenous glutamate release in early stages of an OGD protocol resulting in a decrease in evoked synaptic transmission. Furthermore, modulating mGlu_4_ activity with active *trans*-OptoGluNAM4.1 can influence the time to onset of synaptic responses to ODG.

## Discussion

Research into mGlu_4_ receptor physiology has been hampered by two main obstacles. From a purely pharmacological view-point, it has been exceedingly difficult to develop subtype specific orthosteric agonists and antagonists for mGlu receptors. This is related to the fact that the orthosteric glutamate binding site located in the Venus flytrap extracellular domain of the receptor is highly conserved among all subtypes of the same receptor group (Parmentier et al., 2000). While progress has been made concerning mGlu_4_ specific orthosteric agonists (Selvam et al., 2010, 2018; Zussy et al., 2016), specific antagonists are seriously lacking and as such, recent efforts have been directed toward the development of allosteric modulators for these receptors (Flor and Acher, 2012). Another important question concerning mGlu_4_ receptor function pertains to the role that these receptors play in synaptic transmission, and particularly in the cerebellar cortex. mGlu_4_ receptors are highly expressed in this structure (Corti et al., 2002) and have been localized to the pre-synaptic active zone of PF-PC synapses (Mateos et al., 1998, 1999). While these receptors are readily activated pharmacologically, leading to a reduction in glutamate release from PF terminals (Daniel and Crepel, 2001; Abitbol et al., 2008, 2012; Chardonnet et al., 2017), it has been difficult to demonstrate a role for mGlu_4_ as an autoreceptor in the control of glutamatergic synaptic transmission under near physiological conditions. For example, in acute cerebellar slice preparations Lorez et al. (2003) reported that while prolonged high frequency electrical stimulation of PFs revealed short-lived mGlu_4_ receptor activation by endogenously released glutamate, the degree of this activation was modest compared to that obtained with non-saturating concentrations the group III agonist, L-AP4. As such, it appears that in *ex vivo* preparations at least, PF presynaptic mGlu_4_ receptors are not readily activated by endogenous glutamate released by low frequency electrical stimulation of these same fibers.

In this study, we highlight two important findings. First we show that a recently developed photo-switchable NAM specific for mGlu_4_ receptors, OptoGluNAM4.1 (Rovira et al., 2016), can modulate the activity of native mGlu_4_ receptors at the PF-PC synapse in acute cerebellar slice preparations. It should be noted that this NAM does not completely abolish L-AP4 effects suggesting that OptoGluNAM4.1 only partly blocks mGlu_4_ receptor activity. These findings are consistent with those obtained in cell assays by Rovira et al. (2016). Indeed, they too observed only partial blockade of orthosteric agonist induced mGlu_4_ receptor activity with micromolar concentrations of OptoGluNAM4.1. The second and perhaps the most interesting finding in this study is that PF mGlu_4_ receptors are activated by endogenous glutamate released during the first stages of OGD, an experimental model for brain ischemia.

### OptoGluNAM4.1 Is Functional on Native mGlu_4_ Receptors in Rodent Cerebellar Slices

The search for specific agonists and antagonists for mGlu receptors has been boosted by recent technology that has given rise to photo-isomeric molecules that can be repeatedly switched between their active and inactive configurations, with real time-control, using light (Lerch et al., 2016). Such advances have led to the development of allosteric modulators of mGlu receptors such as Alloswitch-1, a NAM that is specific for mGlu_5_ receptors (Pittolo et al., 2014), Optogluram, a mGlu_4_ specific PAM (Zussy et al., 2016), and OptoGluNAM4.1, a NAM that acts on mGlu_4_ receptors, but also to a lesser degree on mGlu_7_ receptors (Rovira et al., 2016). OptoGluNAM4.1 is a photochromic molecule that is maintained in its mGlu_4_ active (*trans*) isoform in the dark and can be switched to its mGlu_4_ inactive (*cis*) isoform by light emitted in the range of 420-460 nm (blue light). Due the presence of a fast-relaxing azobenzene group, this molecule quickly reverts from its inactive to its active isoform when no longer illuminated with blue light (Rovira et al., 2016).

We first verified that OptoGluNAM4.1 was functional on native presynaptic mGlu_4_ receptors located on PFs in rat and mouse cerebellar cortex. These receptors, when activated pharmacologically with the group III mGlu receptor agonist L-AP4, reduce glutamate release from PFs and attenuate glutamatergic transmission at both PF-PC (Daniel and Crepel, 2001; Abitbol et al., 2008, 2012), and PF-molecular layer interneuron (Chardonnet et al., 2017) synapses. We show that the mGlu_4_ active *trans*-OptoGluNAM4.1 isomer reduces the L-AP4 effect on PF-PC synaptic transmission in rodents. These results corroborate those of Rovira et al. (2016), who have shown that active OptoGluNAM4.1 negatively regulates mGlu_4_ receptor activity in HEK293 cells transfected with a chimeric mGlu_4_ receptor, and has an effect in a mGlu_4_-dependent chronic inflammation model of pain in mice as well as in a behavioral assay in transparent zebrafish larvae. While these later data are extremely interesting, the question remains as to whether the effect of OptoGluNAM4.1 in these *in vivo* model systems results from the modulation of only mGlu_4_ receptors, or whether other group III receptor subtypes are also involved. The PF-PC synapse is an ideal model system in which to study mGlu_4_ receptor physiology since we have already shown that mGlu_4_ is the only group III receptor subtype functional at this synapse (Abitbol et al., 2008).

We recorded PF-evoked excitatory postsynaptic currents (PF-EPSCs) from PCs using a paired pulse stimulation paradigm, an experimental protocol that allows quantification not only of PF-EPSC amplitude, but also the amplitude ratio between the two successively evoked responses. The PPR is a measure that is indicative of the probability of neurotransmitter release and changes in this ratio are suggestive of events occurring at the pre-synapse (Zucker and Regehr, 2002; Jackman and Regehr, 2017). We show that OptoGluNAM4.1 acts on pharmacologically activated presynaptic mGlu_4_ receptors since the ensuing reduction in PF-EPSC amplitude was associated with a significant reduction in the PPR of evoked EPSCs. In agreement with previously published reports (Lorez et al., 2003; Abitbol et al., 2008), we show that endogenous glutamate released with paired stimulations of PFs is insufficient to activate presynaptic mGlu_4_ receptors since OptoGluNAM4.1 alone had no effect on PF-EPSCs. In other words, our experiments with OptoGluNAM4.1 failed to reveal tonic activation of native mGlu_4_ receptors. Finally, we saw no effect of OptoGluNAM4.1 in cerebellar slices obtained from mGlu_4_^-/-^ mice either alone, or with L-AP4 demonstrating that in our experiments this photochromic molecule has no discernable effects on synaptic transmission *per se*, other than specific modulation of mGlu_4_ receptor activity.

Thus, by means of a single blue light source, the photo-switchable OptoGluNAM4.1 can negatively modulate native mGlu_4_ receptor activity in brain slices, and this modulation is reversible in a light–dependent manner. These findings support the idea that this opto-pharmacological molecule is a valuable tool to study mGlu_4_ function.

### Allosteric Versus Orthosteric Antagonism of mGlu_4_ Receptor Activity

OptoGluNAM4.1 reduces L-AP4 depression of PF-EPSCs by reducing the activity of pharmacologically activated mGlu_4_ receptors. As such, glutamate release is increased and the PPR is decreased compared to what we see with L-AP4 alone. However, if we compare the effects of OptoGluNAM4.1 with those of the broad-spectrum group III orthosteric antagonist MSOP, some interesting differences come to light. First, the negative modulation of mGlu_4_ receptor activity by OptoGluNAM4.1 is rather modest compared to that produced by MSOP. This is coherent with data presented by Rovira et al. (2016) who show that in cell assays, OptoGluNAM4.1 only partially blocked L-AP4-activated mGlu_4_ receptor activity. Interestingly, the partial blockade of mGlu_4_ receptor activity observed during co-application of L-AP4 and OptoGluNAM4.1 was not associated with any change in PPR. If we consider that mGlu_4_ activation reduces synaptic transmission exclusively by reducing the probability of action potential induced glutamate release, and that OptoGluNAM4.1 only partially blocks receptor activity, then pharmacological activation of these receptors should result in a slight increase in PPR. The fact that we do not observe any change in PPR suggests that the allosteric modulator, OptoGluNAM4.1, when co-applied with the orthosteric agonist L-AP4, may have additional, ligand-independent effects on mGlu_4_ receptor activity.

Nakajima et al. (2009) have proposed that mGlu_4_ may interact with certain proteins belonging to the SNARE complex and as such contribute to the paired-pulse facilitation typical of the parallel fiber – Purkinje cell synapse in a ligand-independent manner. In our previous studies (Ramos et al., 2012; Chardonnet et al., 2017), we show that native mGlu_4_ receptors interact with several presynaptic proteins, including those belonging to the SNARE complex, including Munc18-1, syntaxin and synapsin. The allosteric modulator OptoGluNAM4.1 binds covalently to the mGlu_4_ receptor (Rovira et al., 2016). Perhaps this binding could influence the configuration of the intracellular domain of mGlu_4_ and alter its interaction with these synaptic proteins, in such a way that pharmacological activation of mGlu_4_ by L-AP4 could result in a decrease in evoked EPSC amplitude, but any ensuing change in PPR would absent or much less apparent.

The second difference we observed is that OptoGluNAM4.1 appears to transiently potentiate the amplitude of PF-EPSCs during the early recovery period following co-application with L-AP4 (overshoot around 5 min after the end of the co-application). This transient potentiation was observed in slices obtained from both rats and WT mice. Interestingly, this phenomenon was not observed following co-application of MSOP and L-AP4.

The question arises then as to whether OptoGluNAM4.1 might transiently antagonize mGlu_4_ receptors following their activation by L-AP4. We know from the cell assay study of Rovira et al. (2016) that OptoGluNAM4.1 covalently binds to the receptor. Consequently, conformational changes in G protein-coupled receptors that occur with orthosteric and allosteric ligand binding may have lasting effects on the protein and its downstream targets, even after wash-out of the orthosteric ligand. One could imagine that following activation of mGlu_4_ receptors by L-AP4 in the presence of OptoGluNAM41, in the subsequent absence of L-AP4, OptoGluNAM4.1 could act as a transient inverse agonist, thus decreasing the basal signaling activity of mGlu_4_. This would result in a temporary increase in the amplitude of evoked EPSCs. That this effect would only be revealed after co-binding of OptoGluNAM4.1 and L-AP4 to their respective sites on the protein is supported by the observation that we see no effect of OptoGluNAM4.1 on PF-EPSC amplitude before pharmacological activation of the receptor. Inverse agonist properties of both positive and negative allosteric molecules have been described for various receptors (Wootten et al., 2013). Alternatively, another possible mechanism is that after wash out of L-AP4, mGlu_4_ receptors covalently bound to OptoGluNAM4.1 adopt a specific conformational transition leading to transient decreases in their basal activity. This in turn would then result in a temporary increase in excitatory transmission. This intermediate state of the receptor protein would slowly return to its initial state, explaining why within a few minutes, PF-EPSC amplitudes attain control levels.

### OptoGluNAM4.1 Reveals Endogenous Activation of mGlu_4_ Receptors Under Physiopathological Conditions: During the Early Phases of Simulated Cerebellar Ischemia

Brain ischemia leads to neuronal death, and one of the most important contributing factors to this morbidity is glutamate-toxicity, since during ischemia there is an important accumulation of glutamate in the synaptic space (Choi and Rothman, 1990; Puyal et al., 2013). We used an *ex vivo* model of ischemia, in which we studied neuronal responses of PCs in acute cerebellar slices, that were subjected to controlled periods of OGD. This protocol, widely adopted to investigate cellular responses to ischemia, results in significant increases in synaptic and extra-synaptic glutamate levels, principally due to a functional reversal of astrocyte glutamate transporters (Henrich-Noack et al., 2000; Rossi et al., 2000; Hamann et al., 2005; Domin et al., 2016).

We show that during the early phase of OGD, glutamatergic transmission at the PF-PC synapse is compromised. In fact, after 5–10 min of OGD, there is a progressive reduction in the amplitude of PF–EPSCs and a concomitant increase in the PPR. These data are comparable to those that Henrich-Noack et al. (2000) obtained in acute hippocampal slices. They show that OGD reduces glutamatergic transmission at the Shaffer collateral – CA1 pyramidal cell synapse, an effect mediated by the activation of group III mGlu receptors. As such we hypothesized that mGlu_4_ receptors contribute to the reduction in glutamatergic synaptic transmission at the PF-PC synapse that we observe during the early phase of OGD.

Recent mGlu_4_ receptor research has provided a wealth of knowledge concerning receptor structure, second messenger signaling pathways, cellular and synaptic responses to pharmacological activation of these receptors and speculations on their physiological roles and pharmaceutical potential. However, to this day it has been difficult to demonstrate that presynaptic mGlu_4_ receptors can be activated by endogenous glutamate released from presynaptic terminals. Our experiments with OptoGluNAM4.1 show that in the presence of either the active *trans*-isomer of this molecule (that is selective for mGlu_4_ receptors) or the broad- spectrum group III antagonist, MSOP, the OGD-dependent decrease in PF–EPSCs and the ensuing increase in PPR is significantly reduced compared to values obtained in the presence of OGD alone, or in the presence of inactive *cis*-OptoGluNAM4.1. Furthermore, we demonstrate that the time to onset of the OGD depressive effect on PF–EPSC amplitude depends on the level of activation of mGlu_4_ receptors – when their activity is inhibited in the presence of active OptoGluNAM4.1 or in the presence of MSOP, the time to onset of this depression is increased. It is interesting to note that while down regulating mGlu_4_ activity increases the time to onset to and attenuates the amplitude of the OGD effect on synaptic transmission, the reduction in PF-EPSC amplitude prevails even in the presence of active OptoGluNAM4.1 and MSOP. While there is a consensus that the principal neurotransmitter released into the extracellular space during OGD is glutamate (Rossi et al., 2000; Hamann et al., 2005), it is possible that other neuromodulators such as adenosine could also be released (or formed from extracellular ATP) during these episodes and thus contribute to decreases in glutamate transmission via the activation of adenosine type 1 receptors that are present on this synapse (Wall and Dale, 2007). It has been shown that activation of these receptors reduces glutamate release from PFs leading to the supposition of their neuroprotective role in ischemia and epilepsy (Dale and Frenguelli, 2009).

## Conclusion

In conclusion, we show, for the first time in the cerebellar cortex, that native mGlu_4_ receptors are robustly activated during early phases of OGD. Their activation leads to inhibition of glutamate release by parallel fiber terminals. Thus, these autoreceptors may play a protective role against ischemic brain damage by reducing the effects of excitotoxicity, confirming the previous data obtained in *in vitro* expression systems, primary cell cultures and *in vivo* model systems (Moyanova et al., 2011; Domin et al., 2016).

OptoGluNAM4.1 presents a new and exciting possibility for studying native mGlu_4_ receptor activity under different physio-pathological conditions in brain tissue, without the need of genetic manipulation. Furthermore, OGD, like other experimental paradigms that result in excessive glutamate release, is an interesting and fruitful model in which to study the protective role of mGlu_4_ receptors. However, our results do not suggest that negative allosteric modulation of mGlu_4_ receptor activity by OptoGluNAM4.1 is a potential therapeutic strategy in the treatment of brain ischemia.

## Author Contributions

SB contributed to the conception and design of the study via data acquisition, analysis and interpretation, and revision of the manuscript. RH participated in data acquisition. MG contributed to data interpretation and revision of the manuscript. XR and AL designed and developed OptoGluNAM4.1 with the participation of EM and AT. XR and AL contributed to the conception of the study and the revision of the manuscript. HD and HM contributed to the conception and design of the study, data analysis and interpretation, preparation and revision of the manuscript.

## Conflict of Interest Statement

The authors declare that the research was conducted in the absence of any commercial or financial relationships that could be construed as a potential conflict of interest.

## Supplementary Material

FIGURE S1 Time to onset of depression of evoked EPSCs during OGD is influenced by the activity of mGlu_4_ receptors. The time to onset of the reduction in PF–EPSC amplitude during OGD under 4 experimental conditions: OGD alone (white, *n* = 27), OGD in the presence of MSOP (gray, *n* = 21), OGD in the presence of inactive *cis*-OptoGluNAM4.1 (blue, *n* = 10) and OGD in the presence of active *trans*-OptoGluNAM4.1 (black, *n* = 8). Data are from the same experiments illustrated in Figure 5. Means ± SEM. (^∗^*p* < 0.05).
