# Supplementary figures and images for: A Light-Controlled Allosteric Modulator Unveils a Role for mGlu4 Receptors During Early Stages of Ischemia in the Rodent Cerebellar Cortex
Source: Front Cell Neurosci. 2018 Nov 27;12:449. doi: 10.3389/fncel.2018.00449 (PMC6277836; doi:10.3389/fncel.2018.00449)

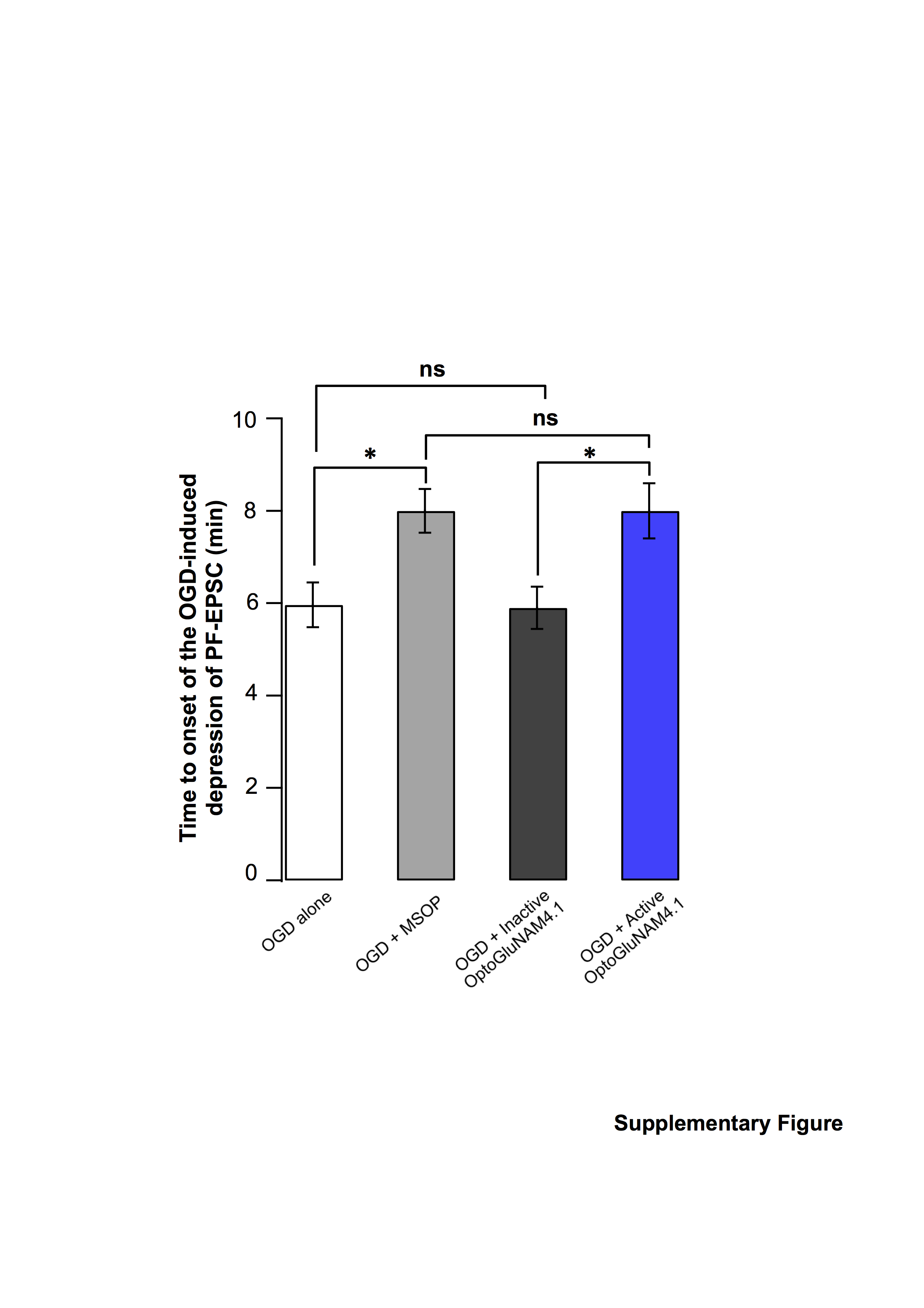

Supplement: FIGURE S1 — Time to onset of depression of evoked EPSCs during OGD is influenced by the activity of mGlu4 receptors. The time to onset of the reduction in PF–EPSC amplitude during OGD under 4 experimental conditions: OGD alone (white, n = 27), OGD in the presence of MSOP (gray, n = 21), OGD in the presence of inactive cis-OptoGluNAM4.1 (blue, n = 10) and OGD in the presence of active trans-OptoGluNAM4.1 (black, n = 8). Data are from the same experiments illustrated in Figure 5. Means ± SEM. (∗p < 0.05). [file Image_1.TIFF]
